# Physical Aggression and Language Ability from 17 to 72 Months: Cross-Lagged Effects in a Population Sample

**DOI:** 10.1371/journal.pone.0112185

**Published:** 2014-11-06

**Authors:** Lisa-Christine Girard, Jean-Baptiste Pingault, Bruno Falissard, Michel Boivin, Ginette Dionne, Richard E. Tremblay

**Affiliations:** 1 Research Unit on Children’s Psychosocial Maladjustment (GRIP), Université de Montreal, Montreal, Quebec, Canada; 2 Paris-Sud Innovation Group in Adolescent Mental Health, Institut National de la Santé et de la Recherche Médicale (INSERM U669), Paris, France; 3 Division of Psychology and Language Sciences, University College London, London, United Kingdom; 4 Faculté de Médecine, Université Paris-Sud, Paris, France; 5 Faculté de Médecine, Université Paris-Descartes, Paris, France; 6 School of Psychology, Université Laval, Quebec City, Quebec, Canada; 7 Institute of Genetic, Neurobiological, and Social Foundations of Child Development, Tomsk State University, Russian Federation, Tomsk, Russia; 8 Departments of Pediatrics and Psychology, Université de Montreal, Montreal, Quebec, Canada; 9 School of Public Health, Physiotherapy, and Population Sciences, University College Dublin, Dublin, Ireland; Georgia State University, United States of America

## Abstract

**Background:**

Does poor language ability in early childhood increase the likelihood of physical aggression or is language ability delayed by frequent physical aggression? This study examined the longitudinal associations between physical aggression and language ability from toddlerhood to early childhood in a population sample while controlling for parenting behaviours, non-verbal intellectual functioning, and children’s sex.

**Methods:**

Children enrolled in the Quebec Longitudinal Study of Child Development (QLSCD) (N = 2, 057) were assessed longitudinally from 17 to 72 months via parent reports and standardized assessments.

**Results:**

The cross-lagged models revealed modest reciprocal associations between physical aggression and language performance from 17 to 41 months but not thereafter.

**Conclusions:**

Significant associations between physical aggression and poor language ability are minimal and limited to the period when physical aggression and language performance are both substantially increasing. During that period parenting behaviours may play an important role in supporting language ability while reducing the frequency of physical aggression. Further studies are needed that utilize multiple assessments of physical aggression, assess multiple domains of language abilities, and that examine the potential mediating role of parenting behaviours between 12 and 48 months.

## Introduction

Physical aggression starts at the end of the first year after birth when children have developed the necessary motor skills to hit, grab, bite, and kick [1]–[3]. The frequency of physical aggression then peaks between 2- and 4-years-of-age; afterwards, a steady decline in physical aggression is typical in most children through to adulthood [4], [5]. This decline coincides with the development of higher-level skills in language, perspective taking, impulse control, and emotional regulation [5], [6]. The use of language also starts to develop in the first year after birth and begins with paralinguistic communications such as the use of babbling, gestures, and vocalizations [7]. Between 12 and 24 months children will subsequently start to retain meaning in language, undergo a large vocabulary spurt, and begin to combine words [8]–[10]. Vocabulary size by the age of two has been found to be a stable predictor of later language ability [8], [10].

### Prospective Associations between Physical Aggression and Language

Studies with clinical samples have reported an association between externalizing problems such as aggressive behaviour and language ability, however the children in these studies were selected because of varying degrees of behaviour problems or language impairment [11]–[17]. The use of clinical samples may result in inflated estimates of associations found due to co-occurring disorders [18], and therefore may not accurately represent the association in the general population. Further, the specific association between physical aggression and language ability has been understudied as many studies have focused on aggregate measures of externalizing behaviours [11]–[13], [15], [17], [19]–[21]. Unfortunately, the use of longitudinal studies examining physical aggression with population samples commencing in the toddler years (around the onset of physically aggressive behaviours and language), are scarce. Indeed, the majority of studies that used non-clinical samples focused on middle childhood and early adolescence (e.g., [22], [23]). In these studies, associations between lower language ability to subsequent increases in physically aggressive behaviours were observed over time. These findings suggest that even within nonclinical populations, children with lower language abilities may present with increased risk for engagement in future physical aggression. Support for the long-term efficacy of early prevention efforts has been noted in the literature [24], [25], suggesting the importance of examining these associations when they may first occur (i.e., in the toddler years).

To the best of our knowledge there have only been two studies to date conducted with population samples as early as two years of age examining the association between physical aggression specifically, and language ability [26], [27], and in these studies the association was not assessed longitudinally. Support was however found for associations between expressive language and physical aggression and receptive language and physical aggression. This suggests that in early development, both expressive and receptive language ability are associated with physical aggression. A third study was recently conducted examining the longitudinal associations between anger expression and language skills in toddlerhood [28]. The results revealed that toddlers with better early language skills at 18 months were less likely to engage in later anger expressions at 48 months. Less support surrounding the inverse association (i.e., anger expression to language) was found. While anger expression may be a catalyst to engagement in consequent displays of physical aggression, physical aggression was not specifically examined in this study.

Both physical aggression and poor language ability have been associated with long-term maladaptive outcomes [13], [29], thus a better understanding of the association starting in the toddler years is warranted. This may for example, help in designing prevention efforts targeting the initial problem whereby reducing the emergence of consequent problematic functioning in the other domain of children’s development. In the current study, we focus on the associations between physical aggression and language abilities in a typically developing population-based cohort sample from 17–72 months.

### Theoretical Models of Aggression and Language

Three models have been used to explain the association between externalizing behaviours such as aggression and language development. The first model assumes poor language leads to the onset of aggressive behaviours because the ability to communicate in social situations is impaired [19], [22], [23], [26]. This can result in frustration and the use of aggression as an alternative tool for communication. Further, poor language skills can impede on a child’s ability to effectively resolve conflicts during social situations thereby increasing the likelihood of the use of aggressive behaviours [30]–[32]. This direction of association may be particularly salient around the ages of four to five when children enter into formal schooling and have more exposure to peer interactions. Longitudinal studies with both clinical and nonclinical samples have supported the association from poor language to increased externalizing problems such as aggression from the ages of five into adolescence and adulthood [11]–[14], [23].

The second model posits that aggression can lead to delayed language development [16], [20], [33]. One perspective to support this model suggests that when children engage in high levels of aggressive behaviours they spend less time attending to the verbal stimuli in their environment [26]. This inattention may limit their learning opportunities to develop language skills. Additionally, parents who are always attending to aggressive children’s behaviour may focus less on providing a rich language model for their child and may focus more on ways of curbing engagement in physical aggression [26]. It may then be more likely that this association would be observed in the toddler years when physical aggression is at its peak and when a rich language model is needed and critical for language growth [1], [2], [34].

The third model posits that there is some underlying third variable implicated in the association between aggression and language. While not exhaustive, examples of variables previously implicated would include parenting behaviours such as positive, and harsh parenting [35]–[43], in addition to children’s intellectual functioning [9], [20], [21], [44]–[46]. The association between parenting behaviours and subsequent externalizing behaviours such as aggression have been well documented whereby harsh parenting has been found to be positively associated with increased engagement in aggressive behaviours and positive parenting such as warmth and sensitivity have been shown to protect against and reduce engagement in these behaviours [35], [36], [39]. Parent-child interactions provide children with working knowledge of the social world and model appropriate behavioural response (i.e., social learning theory) [32]. Thus, it is not surprising that the behavioural responses of parents transmit to the types of behaviours that children may then in turn enact.

Moreover, parenting behaviours have also been shown to impact upon the acquisition and growth of language ability over time [34], [37], [38], [42]. For example, positive parenting in particular can facilitate an environment in which children are exposed to greater frequencies of language-based exchanges. This in turn may provide children with additional supports for learning to express their needs and understand others through a positive, supportive, and reinforcing environment. Conversely, family environments characterized by harsh parenting may limit the opportunity for language exchanges between parent and child and consequently negatively impact upon language learning [34], [40]. The toddler years are marked by developmental transitions across multiple domains and thus parenting behaviours may be particularly salient to both children’s linguistic and social development during this time. Further, before children enter into formal schooling, parent-child interactions comprise the most frequent opportunities for learning. Given the associations found in the literature among parenting behaviours, engagement in physical aggression, and language learning, it was important to control for possible effects of parenting behaviours.

### Objectives

The overall objective of the current study was to examine the longitudinal associations between physical aggression and language ability over and above the contributions of parental warmth, consistency, punitive parenting, children’s non-verbal intellectual functioning, and sex. More specifically, we attempted to identify the best model fit with respect to directionality of the associations between physical aggression and language ability from 17–72 months. We also examined whether the direction of the associations varied across differing stages of development. Based on the theoretical models put forth and a review of the literature examining behaviour problems and language, we might expect to observe changes in the associations across time. That is, in the toddler years increased physical aggression may lead to lower language ability from 17–41 months whereas from 41–72 months the inverse association may be more plausible. However, in the current study no specific predictions were made as there is still mixed support in the literature surrounding directionality, limited studies that have looked at this association longitudinally commencing as early as two years of age, and few studies that have examined physical aggression specifically.

## Methods

### Participants

Children taking part in the current study were enrolled in the Quebec Longitudinal Study of Child Development (QLSCD), a cohort sample comprised of singletons born in Quebec, Canada between 1997 and 1998. The QLSCD participants were drawn from the Quebec Birth Registry using stratification procedures that are documented extensively elsewhere [47]. Children (N = 2,057) were assessed via parent report at 17, 29, 41, 60, and 72 months. At 41, 60, and 72 months, standardized assessments were also conducted. Both Francophone and Anglophone versions of the parent reports and standardized assessments were utilized given the community make up in Quebec. In the current sample, 11.13% of mothers were of immigrant status. Eighty-two percent of mothers were Native French speakers, 10% were Native English speakers, 2% spoke both French and English, and 2% spoke French, English, and an additional language. Eighty-nine percent of children were French speakers. Eighteen percent of mothers were employed and 19.11% of children were living in a non-intact family structure. The sample was comprised of 1043 boys and 1014 girls. All data were collected during home visits and informed written consent was obtained from the primary caregiver at each assessment period. Ethics approval was obtained and approved by the Québec Institute of Statistics’ Ethics Committee.

### Measures

#### Outcomes

Physical aggression was assessed via parent report. Items on the physical aggression scale were taken from a variety of behaviour rating scales (Achenbach-Child Behavior Checklist; Preschool Behavior Questionnaire; Children’s Behaviour Questionnaire) [48]–[51], which have all been well validated in the literature. At 17 and 29 months 12 items were selected with examples of items including (a) takes things away from others, (b) pushes others, (c) kicks others, and (d) hits others. At 41, 60, and 72 months items included (a) hits, kicks, bites others; (b) gets into fights with other children, and (c) bullies others. These items were selected as they have been found to be reliable in assessing physical aggression in childhood [4], [52]. Parents reported on the frequency of aggression items as never (0), sometimes (1), or often (2). Cronbach’s alpha for physical aggression items at 17, 29, 41, 60, and 72 months was .78, .81, .72, .73, .70, respectively.

Children’s language ability at 17 and 29 months was assessed via parent report using items from the McArthur Communicative Development Inventory-Short form (MCDI), which is normed for toddlers between 16 and 30 months [53]. The MCDI is one of the most widely used assessments of toddlers’ language ability and a stable predictor of language development longitudinally [10]. Additionally, at 29 months parents completed a 100-word checklist capturing words that children can both produce and understand. At 41, 60, and 72 months, children’s language was assessed using the Peabody Picture Vocabulary Test (PPVT) [54], a standardized measure of receptive language normed for children 2∶6 and older. The psychometric properties of the PPVT are excellent (i.e., Cronbach’s alpha in the current sample ranges from .93–.98) and have been well validated. Children’s standardized scores were utilized. The use of differing assessments across time for physical aggression and language in the current study was in part the result of applicability to specific points of development and is discussed further in the limitations section.

#### Covariates

Children’s non-verbal intellectual ability was first assessed at 41 months using the Wechsler Preschool and Primary Scale of Intelligence (WPPSI) [55]. Because the WPPSI is a standardized measure of intelligence that is normed for children ages 2∶6–7∶3, we were unable to administer this assessment before 41 months. The block design subtest was utilized. Cronbach’s alpha for this subtest is reported as .89.

Parental warmth, consistency, and punitive parenting were assessed via parent reports when children were 29 months using items from the Parent Practices Scale [56]. This was the first assessment period when information on all three scales was available. Parents were asked to rate the frequency for which they engaged in select behaviours over the course of the previous 12 months utilizing a multiple choice format. Both validity and reliability of this scale have been documented in the literature [56].

### Statistical Analysis

Cross-lagged models were utilized in the current study. While causality cannot be directly inferred through the use of cross-lagged models, this statistical approach was employed as it allows for examination of longitudinal bi-directional paths between physical aggression and language ability. Cross-lagged models have been under-utilized in research examining the associations between physical aggression and language ability.

The chi-square test to assess model fit is presented for each model. Since the chi-square test is likely to be significant with large samples [57], we also provide approximate indices of fit including the root mean square error of approximation (RMSEA) [58] and the comparative fit index (CFI) [59]. MacCallum et al. [60] have suggested a cutoff value below .08 for the RMSEA as representing a good model fit. With respect to the CFI, Hu & Bentler [61] have suggested a cutoff of equal to or higher than .95 as representing a good model fit. The CFI was selected in addition to the commonly reported RMSEA, as the CFI is a measure of fit least affected by the sample size [62].

Cross-lagged models were estimated using Mplus version 6.11 [63]. All physical aggression and language variables were treated as dependent variables allowing for the possibility of reciprocal changes in the association at differing stages of development rather than imposing directionality in the model. All missing data were treated as missing at random using Full Information Maximum Likelihood (FIML). Standardized Betas (β) are presented for each model. Three different paths of associations were evaluated, namely, auto-regressive (e.g., from aggression at 17 months to aggression at 72 months), concurrent (e.g., between aggression and language at 17 months), and cross-lagged (e.g., from aggression at 17 months to language at 29 months).

## Results

Table 1 presents the means and standard deviations of both physical aggression and language variables from 17–72 months. Bivariate correlations between physical aggression and language variables are presented in Table 2. Inspection of the bivariate correlations reveals marginally significant associations between physical aggression and language ability at varying times, consistent with previous studies of normally developing children. Notably, physical aggression at 29 months is associated with children’s language ability at each assessment period. All significant associations are in the expected direction whereby higher physical aggression is associated with lower language ability, albeit the effect size is small.

**Table 1:** Mean Scores and Standard Deviations of Physical Aggression and Language Ability from 17–72 Months.

Age		Aggression	Language
**17 Months**	Mean (SD) Min–Max	1.33 (1.26) 0–8	1.70 (0.72) 0–3
**29 Months**	Mean (SD) Min–Max	1.74 (1.40) 0–10	40.71 (10.01) 0–50
**41 Months**	Mean (SD) Min–Max	2.23 (1.54) 0–9	30.00 (15.53) 2–91
**60 Months**	Mean (SD) Min–Max	2.00 (1.60) 0–9	67.01 (18.93) 2–119
**72 Months**	Mean (SD) Min–Max	1.90 (1.60) 0–9	80.40 (17.15) 0–130

Note. Composite scores of the frequency of physical aggression items via parent report are presented here. Language at 17 and 29 months was assessed via parent report using the McArthur Communicative Development Inventory-Short form. At 41–72 months language was assessed using the Peabody Picture Vocabulary Test.

**Table 2:** Bivariate Correlations between Physical Aggression and Language Ability from 17–72 Months.

	Agg 17 m	Agg 29 m	Agg 41 m	Agg 60	Lang 17 m	Lang 29 m	Lang 41 m	Lang 60 m	Lang 72 m
**Agg 17 m**					*r* = –.02 *ns*	*r* = –.01 *ns*	*r* = –.08 *p* = .00	*r* = –.02 *ns*	*r* = –.06 *p* = .04
**Agg 29 m**	*r* = .51 *p* = .00				*r* = –.05 *p* = .02	*r* = –.08 *p* = .00	*r* = –.09 *p* = .00	*r* = –.07 *p* = .03	*r* = –.08 *p* = .00
**Agg 41 m**	*r* = .37 *p* = .00	*r* = .52 *p* = .00			*r* = –.02 *ns*	*r* = –.01 *ns*	*r* = –.05 *p* = .04	*r* = –.05 *ns*	*r* = –.00 *ns*
**Agg 60 m**	*r* = .24 *p* = .00	*r* = .39 *p* = .00	*r* = .56 *p* = .00		*r* = –.02 *ns*	*r* = –.03 *ns*	*r* = –.05 *ns*	*r* = –.03 *ns*	*r* = –.01 *ns*
**Agg 72 m**	*r* = .24 *p* = .00	*r* = .37 *p* = .00	*r* = .50 *p* = .00	*r* = .62 *p* = .00	*r* = –.03 *ns*	*r* = –.03 *ns*	*r* = –.05 *p* = .04	*r* = –.04 *ns*	*r* = –.04 *ns*
**Lang 29 m**					*r* = .34 *p* = .00				
**Lang 41 m**					*r* = .13 *p* = .00	*r* = .42 *p* = .00			
**Lang 60 m**					*r* = .10 *p* = .00	*r* = .40 *p* = .00	*r* = .57 *p* = .00		
**Lang 72 m**					*r* = .15 *p* = .00	*r* = .39 *p* = .00	*r* = .51 *p* = .00	*r* = .72 *p* = .00	

### Bivariate Cross-lagged Model: Physical Aggression and Language from 17–72 Months

**Figure 1:**
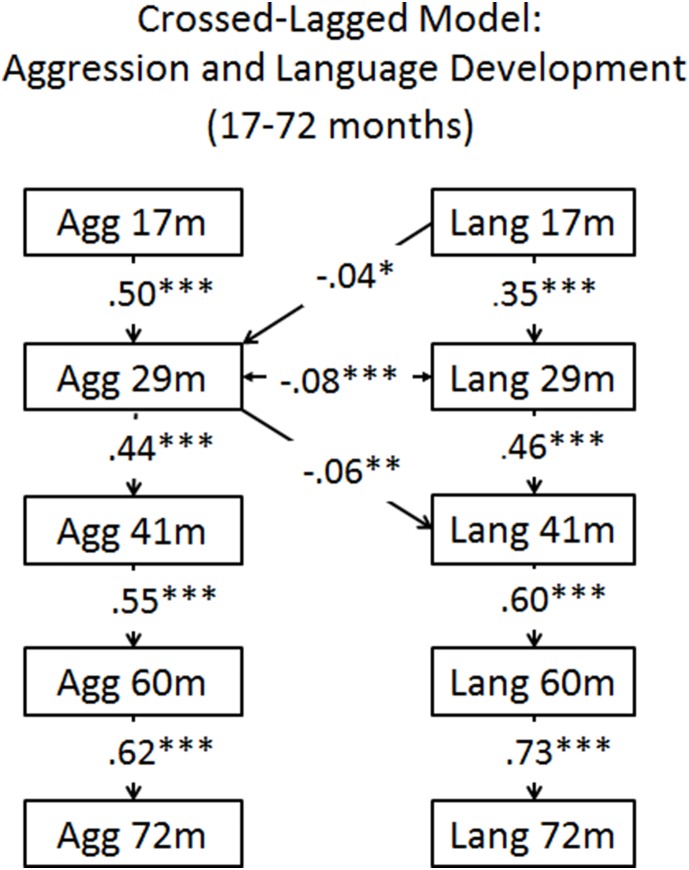
Crossed-lagged model: Aggression and language development from 17–72 months. Full Information Maximum Likelihood used. Note: ***Significant at the .001 level. **Significant at the .010 level. *Significant at the .050 level.

Overall model fit for the first model without covariates was acceptable, *x* ^2^ (23) = 238.01, *p*<.001; RMSEA = .07; RMSEA CI_90_ = 0.060–0.075; CFI = .95. Auto-regressive paths revealed medium to large significant positive associations for both physical aggression and language variables (i.e., β = .44–.62, and .35–.73, *p* = <.001, respectively). In this model, only one significant negative concurrent path was found between physical aggression and language ability at 29 months, (i.e., β* = *–.08, *p* = <.001). The results of the cross-lagged paths revealed two significant associations. First, lower language ability at 17 months was associated with more frequent physical aggression at 29 months (i.e., β* = *–.04, *p* = .049). Second, more frequent physical aggression at 29 months was significantly associated with lower language ability at 41 months (β* = *–.06, *p* = .002). No other cross-lagged associations were found (see Figure 1).

### Cross-lagged Model with Covariates Entered

**Figure 2:**
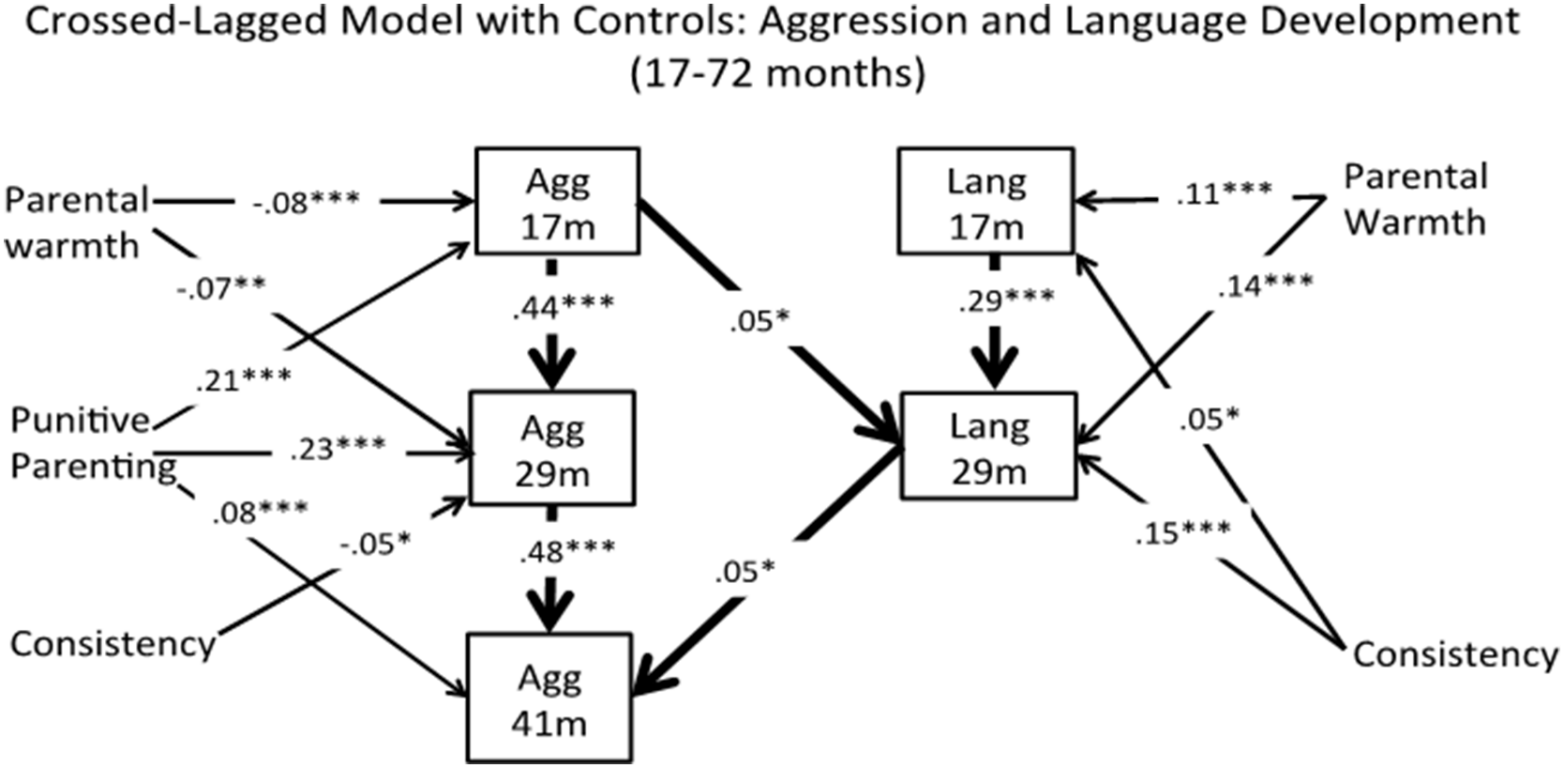
Crossed-lagged model with controls: Aggression and language development from 17–72 months. Full Information Maximum Likelihood used. Note: ***Significant at the .001 level. **Significant at the .010 level. *Significant at the .050 level. For visual simplicity of the model we report here only significant associations up to 41 months as no significant cross-lagged associations between aggression and language were found thereafter. The full model may be requested from authors. We also note that while not included in the visual model for simplicity, sex was associated with Aggression at 17 and 29 months (i.e., β = .05, *p* = .024, β = .05, *p* = .017, respectively), and with Language at 17 and 29 months (i.e., β = –.12, *p* = .000, and β = –.09, *p* = .000, respectively). Finally, all parenting factors were collected at 29 months.

It is possible that within-child factors and parenting behaviours contribute to both the development of children’s physical aggression and language ability and may consequently impact on the presentation of associations over time. We therefore examined a second cross-lagged model of physical aggression and language ability controlling for parental warmth, consistency, and punitive parenting, non-verbal intellectual ability, and sex. Model results are presented in Figure 2. Overall model fit was acceptable, *x* ^2^ (28) = 286.76, *p*<.001; RMSEA = .07; RMSEA CI_90_ = 0.060–0.074; CFI = .95. Auto-regressive paths for physical aggression and language ability were once again significantly associated over time (i.e., β = .44–.59, and .29–.70, *p* = <.001, respectively). No concurrent associations between physical aggression and language abilities were found once covariates were entered into the model. Cross-lagged associations revealed that physical aggression at 17 months was now positively associated with language ability at 29 months (β = .05, *p* = .019). Additionally, children’s language ability at 29 months was positively associated with aggression at 41 months (β = .05, *p* = .023) indicating possible suppression effects of covariates. No other cross-lagged associations were found.

In the current sample, boys were rated higher as compared to girls on physical aggression at all assessment periods with the exception of 41 months (β* = *.05–.06, *p* = <.050). Boys also had lower ratings on the language measures as compared to girls at 17 and 29 months (β* = *–.11, –.09, *p* = <.001), however no significant differences were found thereafter. Interestingly, children’s non-verbal intellectual ability was never found to be significantly associated with physical aggression at any stage of development, however was positively associated with language at 29, 41, 60, and 72 months (β* = *.07–.25, *p* = <.010). With respect to parenting behaviours, parental warmth was negatively associated with physical aggression at 17 and 29 months (β* = *–.08, –.06 *p* = <.010), and positively related to language ability at all times (β = .06 –.14, *p* = <.050) with the exception of 60 months. Consistency was negatively associated with physical aggression at 29 months (β* = *–.05, *p* = .014), and positively associated with language ability at 17, 29, and 41 months (β* = *.05–.08, *p* = <.050), but not thereafter. Finally, punitive parenting was consistently positively associated with children’s physical aggression (β* = *.08–.22, *p* = <.001) but never with language (see Figure 2).

## Discussion

The objectives of the current study were to examine the putative associations between physical aggression and language ability in infancy and early childhood while controlling for parenting behaviours, children’s non-verbal intellectual ability, and sex. The results contribute to the literature in important ways. First, to the best of our knowledge this is the first study that examined the developmental associations between physical aggression and language ability from 17 months onwards in a population cohort, and the results demonstrate that associations present as early as between 17 and 29 months. Second, cross-lagged models were utilized as this is a more stringent form of analysis that does not impose assumptions of directionality, and the results of the current study support reciprocal changes in the associations across time. Finally, controlling for the role of parenting behaviours and within-child factors led to different patterns of associations.

Results of the cross-lagged model without covariates revealed reciprocal negative associations between children’s physical aggression and language ability from 17 to 41 months. That is, toddlers with lower language ability at 17 months were rated higher on physical aggression at 29 months, lower language ability at 29 months was associated with higher physical aggression at 29 months, and higher physical aggression at 29 months in turn was associated with lower language at 41 months. However, in line with previous studies, the effect sizes were small [14], [26], [64]. No significant associations were found thereafter which is in contrast to previous studies with older samples of children. It is possible that associations were only observed between 17 and 41 months, as this is a developmental period marked by high transitions for both social behavioural and language development. Thus, our results with a large population sample lend minimal support to the various hypotheses that physical aggression and language ability have important impacts on one another during infancy and early childhood.

When parenting behaviours, non-verbal intellectual ability, and sex were entered into the model, the associations between physical aggression and language ability between 17 and 41 months changed from negative to positive associations. Further, different directions of associations were found whereby higher physical aggression at 17 months was associated with language ability at 29 months and in turn language ability at 29 months was associated with physical aggression at 41 months. Theoretically, it would not be expected that higher physical aggression would lend to increased language ability over time as physical aggression is often used as an alternative form of communication when language, emotional regulation, and social maturity are lacking. Further, engagement in higher physical aggression may distract from the language-learning environment as a greater focus of parents may be placed on the reduction of these behaviours. It also seems improbable that better language ability would lead to increased physical aggression over time as language ability can facilitate the resolution of conflict during social interactions. Of note is that effect sizes for these reversed associations were also small.

Thus, two possible explanations for these findings are presented. First, the change in direction of associations following the addition of covariates into the model may suggest that suppression effects and/or mediation are occurring. For example, examination of covariates in Figure 2 are suggestive of punitive parenting having the strongest effect on increased physical aggression and parental warmth on both lower physical aggression and better language ability during the times in which significant associations are found. However, in the current study suppression and mediation were not directly tested as a result of temporal issues with covariates. That is, our first measures of parenting behaviours were collected at 29 months, which is 12 months after the first assessments of physical aggression and language ability. This limits our ability to directly test whether initial parenting behaviours influence children’s physical aggression and language ability between 17 and 29 months or whether parents’ behaviours are influenced by children’s physical aggression and language ability during this time. Although we are limited in our ability to test these directional associations, it is highly probable that these associations are in fact dynamic processes. There is a clear need for a better understanding of how parenting behaviours are implicated in the context of the cross-lagged associations between physical aggression and language ability. In the current study, only direct effects of parenting rather than interaction effects are possible to deduce. It is therefore recommended that further studies be conducted that directly test for possible suppression and mediation effects. This can be done by utilizing multiple assessments of physical aggression, language ability, and parenting behaviours between 12 and 48 months, the period during which children substantially increase the frequency of their physical aggression as well as their language proficiency. A related explanation may be that while significant associations between physical aggression and language ability were found, physical aggression and language ability may be parallel rather than predictive processes that are affected by other factors in children’s development.

The current work gives some support to an effect of parenting behaviours in the associations between physical aggression and language ability in early childhood, although the exact nature remains unclear. More population studies are needed to further examine parenting behaviours in addition to other possible variables that may contribute to these associations, as the controls used in the current study were by no means exhaustive. Such studies should at least be genetically informative and ideally assess sibling and peer contributions to both language and physical aggression. Future studies should also assess multiple domains of language ability in addition to expressive and receptive language from infancy to early childhood. Analyzing separate models for physical aggression and specific types of language ability (e.g., expressive, receptive, pragmatic) will provide a better understanding of the language processes driving the observed associations.

### Limitations of the Present Study

While all language assessments in this study were previously well validated, they differed across development as a result of applicability for different age groups. However, the strong auto-regressive paths between language measures at successive time points would suggest that while different, the measures of language are tapping closely related skills. Therefore, if associations between physical aggression and language ability exist in early childhood, we would expect that they would be revealed in the current study. Given the fact that we observed associations between aggression and language up to 41 months only, and that language at 29 months is consistently implicated, it may be that the association between physical aggression and language is contingent upon the inclusion of both expressive and receptive language. Additionally, we did not assess other areas of language ability such as pragmatic language, semantics, or syntax, which may also be implicated in the association with physical aggression.

A second limitation is related to parenting variables. Not all parenting variables were available at 17 months and thus the parenting variables that were collected at 29 months were used. At 29 months parents were asked to rate frequencies over the past 12 months, which theoretically would be representative of parenting behaviours starting from 17 months. However, given that parents were asked to retrospectively recall their behaviour over the previous 12 months, there may be some effects of recall bias present. Additionally, combining parent report with direct observations of parent-child interactions may provide a more robust measure of parenting behaviours. Thus, caution must be taken in interpretation of the current study results until replication is found. Finally, cognitive assessments were available only at 41 months, whereby preventing us from assessing the impact of intellectual functioning prior to 41 months, the period in which significant associations between aggression and language were observed.

## Overall Conclusions

Overall results of the current study suggest that in a population cohort, there is modest support for a direct association between physical aggression and language ability in infancy and early childhood. While significant concurrent and cross-lagged paths are observed in the simple model, the effect sizes are weak. Previous studies that found stronger associations differed from the current study in that they used clinical samples, older children, and aggregated measures of problem behaviour. Results also raise the possibility that parenting behaviours influence the development of the association between children’s physical aggression and language from 17–41 months, a developmental period when physical aggression and language performance are rapidly increasing.
